# Animal models for oral transmission of *Listeria monocytogenes*

**DOI:** 10.3389/fcimb.2014.00015

**Published:** 2014-02-11

**Authors:** Sarah E. F. D'Orazio

**Affiliations:** Department of Microbiology, Immunology and Molecular Genetics, University of KentuckyLexington, KY, USA

**Keywords:** intragastric, food borne, listeriosis, intracellular pathogen, mouse, guinea pig

## Abstract

*Listeria monocytogenes* has been recognized as a food borne pathogen in humans since the 1980s, but we still understand very little about oral transmission of *L. monocytogenes* or the host factors that determine susceptibility to gastrointestinal infection, due to the lack of an appropriate small animal model of oral listeriosis. Early feeding trials suggested that many animals were highly resistant to oral infection, and the more reproducible intravenous or intraperitoneal routes of inoculation soon came to be favored. There are a fair number of previously published studies using an oral infection route, but the work varies widely in terms of bacterial strain choice, the methods used for oral transmission, and various manipulations used to enhance infectivity. This mini review summarizes the published literature using oral routes of *L. monocytogenes* infection and highlights recent technological advances that make oral infection a more attractive model system.

## Listeriosis is a food borne infection in humans

Food borne transmission of *L. monocytogenes* occurs when ready-to-eat foods become contaminated during processing (Carpentier and Cerf, 2011), the bacteria multiply during refrigeration (Tasara and Stephan, 2006), and the food is consumed without heating (Rocourt et al., 2003). The actual incidence of intestinal infection is unknown, but the attack rate for heavily contaminated foods is probably high (Dalton et al., 1997; Ooi and Lorber, 2005). Most patients do not seek medical treatment for self-limiting gastroenteritis, and *L. monocytogenes* is not readily isolated from stool without the use of specific enrichment broth (Scallan et al., 2011). After a short incubation, *L. monocytogenes* can spread systemically and cross the blood-brain barrier or the placenta. A recent analysis of several outbreaks found that gastrointestinal symptoms appeared within 24 h of ingestion and bacteremia occurred within 2 days (Goulet et al., 2013). However, it took an average of 9 days for CNS symptoms to emerge, and much longer (17–67 days) for pregnancy-associated cases to be reported. Even with antibiotic treatment, the invasive form of listeriosis has a high mortality rate, particularly in immune-compromised individuals (Wing and Gregory, 2002).

An ideal animal model of listeriosis should mimic all phases of the human disease. Oral transmission is preferred, to allow investigation of environmental factors that promote infectivity, host resistance mechanisms in the gut, and bacterial virulence properties that promote colonization and subsequent dissemination. Only a small subset of humans exposed to *L. monocytogenes* develop severe gastroenteritis, bacteremia, or meningoencephalitis; thus, systemic spread should be a rate-limiting step. Ideally, resistant animals should be able to rapidly contain bacterial growth, and there should be a lag period before *L. monocytogenes* cross the blood-brain barrier or the maternal-fetal interface in susceptible animals.

## Choice of animal

Most existing models of listeriosis have caveats that either pose significant technical challenges or limit their ability to closely mimic human disease. One such limitation is the high bacterial dose required to establish infection in laboratory animals. The infectious dose for humans has not been clearly established, but was estimated to be 10^6^–10^7^ CFU (FAO/WHO, 2004; Smith et al., 2008). Typically, much larger inocula (10^8^–10^10^CFU) are needed for experimental infections. *L. monocytogenes* are not particularly acid-tolerant, and a large portion of any ingested dose is likely to be killed in the stomach. Comparative studies to address potential differences between humans and experimental animals in stomach pH, bile content, or gastric enzyme composition are still needed. It is also possible that prolonged gastrointestinal infection only occurs in otherwise healthy individuals when very large doses are ingested. In that case, the range of inocula used in immune competent laboratory animals may, in fact, closely mimic the human condition.

Another issue is the species specificity of the bacterial surface proteins InlA and InlB for their corresponding receptors expressed on host cells (Mengaud et al., 1996; Shen et al., 2000). Early work indicated that InlA was important for uptake in epithelial cells (Gaillard et al., 1991; Lecuit et al., 1997), and InlB was needed for invasion of endothelial cells (Greiffenberg et al., 1998; Parida et al., 1998). However, more recent studies suggest that these two receptors may work together to promote efficient uptake of *L. monocytogenes* into non-phagocytic cells (Pentecost et al., 2010; Grundler et al., 2013). InlA has a high affinity for human E-cadherin, and the human Met protein serves as a receptor for InlB, so the full complement of surface protein interactions is available to promote uptake of *L. monocytogenes* during human infections (Lecuit et al., 1999). As detailed below, most of the commonly used animal models feature a low affinity interaction for either InlA or InlB, and this represents a major limitation for the use of each model. Intestinal infection and feto-placental transfer can still occur in these animals, suggesting that other uptake mechanisms may compensate for the lack of this pathway, but it is not yet clear how accurately these systems model human infections.

### Rabbits, sheep, and goats

*L. monocytogenes* was first identified in 1926 as the causative agent of a mononuclear leukocytosis in rabbits (Murray et al., 1926). Until the 1980s, the bacterium was regarded primarily as a veterinary pathogen, with naturally occurring listeriosis found mainly in sheep and other ruminants (Gates et al., 1967). Thus, early research efforts focused on feeding trials to mimic the disease observed in rabbits and sheep (Osebold and Inouye, 1954; Gray et al., 1955). Although some investigators do still use rabbit (Belen Lopez et al., 1993) or goat (Miettinen et al., 1990) models to study oral transmission, most research is now focused on rodent and non-human primate models. For a comprehensive review of infection studies in ruminants and other veterinary models, see Hoelzer et al. (2012).

### Rodents

#### Mice and rats

The small size of rodents makes large-scale experiments feasible, and there is an abundance of commercially available reagents, particularly for mice. Early work suggested that 10^8^–10^10^ CFU were needed to establish intestinal infection, and that intravenous (i.v.) inoculation was more consistently lethal in mice (Audurier et al., 1980; Golnazarian et al., 1989); however, infectious dose is highly dependent on strain background. For example, both A/J and BALB/c/By/J mice are significantly more susceptible than C57BL/6 mice (Czuprynski et al., 2003a; Bou Ghanem et al., 2012; Bergmann et al., 2013) with intestinal colonization requiring as few as 10^6^ CFU. Differences in the microbiota of these mice do not appear to be a key factor, as fecal transplantation between C57BL/6 and BALB/c/By mice did not alter either susceptibility or resistance to orally acquired *L. monocytogenes* (Myers-Morales et al., 2013).

To enhance oral infection in mice, the Lecuit group generated two “humanized” mouse strains that promote interaction between InlA and E-cadherin. In the first, human E-cadherin is ectopically expressed under the control of the iFABP promoter, resulting in dual expression of both mouse and human E-cadherin in cells of the small intestine (Lecuit et al., 2001). The second is a “knock-in” strain with a single amino acid substitution (E16P) that allows murine E-cadherin to serve as a receptor for InlA (Disson et al., 2008). Oral infection studies with E16P mice clearly showed that InlA can enhance colonization in the gut, and this mouse strain represents the best option for mimicking the InlA/InlB mediated invasion events that occur in humans. However, as with most transgenic mice, the knock-in was generated on the highly resistant C57BL/6 background. Although time-consuming and expensive, it would be useful to cross the E16P mutation onto more susceptible strain backgrounds.

#### Guinea pigs

Guinea pigs require doses of 10^8^–10^10^ CFU to achieve intestinal colonization in 3–4 week old animals (Roldgaard et al., 2009; Melton-Witt et al., 2012). Melton-Witt et al. infected guinea pigs with a mixture of 20 signature-tagged strains and found that dissemination from the MLN to the spleen represented a rate-limiting step in the infection, with only 1 in every 100–1000 bacteria making it past this bottleneck (Melton-Witt et al., 2012). Guinea pig E-cadherin serves as a receptor for InlA, but the Met protein in guinea pigs has an amino acid change that prevents optimal interaction with InlB. Thus, InlA/InlB-mediated uptake pathways will not be fully functional in guinea pigs. Despite this limitation, guinea pigs have been favored for use in studying maternal-fetal transmission of *L. monocytogenes*, because the placentas of other rodents do not have the same architecture as humans. Both guinea pigs and humans have hemochorial placentas with only a single layer of trophoblasts separating the maternal and fetal blood supplies during the later stages of pregnancy (Leiser and Kaufmann, 1994). Williams *et al.* have shown that infection of pregnant guinea pigs with oral doses ranging from 10^4^ to 10^8^ CFU resulted in invasion of fetal tissue in approximately half of the fetuses (Williams et al., 2007, 2011).

#### Gerbils

Gerbils do not have a species barrier for either InlA or InlB (Disson et al., 2008), and thus, may be the most physiologically relevant rodent model for listeriosis. Disson et al. orally inoculated gerbils and found significant colonization of both the small and large intestines, and 100% fetal lethality when pregnant females were used (Disson et al., 2008). The inocula used in those studies were 10^9^–10^10^ CFU, and lower doses were not tested. Blanot et al. also showed that gerbils consistently developed rhombencephalitis that closely mimicked human brain infections (Blanot et al., 1997). However, the gerbils were infected via the middle ear, so it is not yet clear whether brain infections occur spontaneously after oral ingestion in gerbils. Unfortunately, a lack of available tools and reagents specific for gerbils has limited enthusiasm for studying host responses in this model.

### Non-human primates

Rare spontaneous outbreaks of listeriosis in non-human primate colonies have resulted in both meningoencephalitis and spontaneous abortion in pregnant females (Smith et al., 2003; Lemoy et al., 2012). Thus, the clinical symptoms of sporadic listeriosis in non-human primates appear to closely mimic human disease. However, non-human primate models have the distinct disadvantage of being expensive, with only small numbers of animals typically available for each study. Nonetheless, the few non-human primate infection studies performed to date have been useful for approximating the infectious dose of *L. monocytogenes*. Farber et al. found that cynomolgus monkeys fed whole milk containing at least 10^7^ CFU of strain Scott A shed *L. monocytogenes* in the feces for 21 days, but only animals given 10^9^ CFU showed clinical signs of disease (septicemia and occasional diarrhea) (Farber et al., 1991). More recently, Smith et al. used a monkey clinical isolate of *L. monocytogenes* to infect pregnant rhesus macaques (Smith et al., 2008). In that study, the overall LD_50_ was estimated to be 10^7^ CFU, but monkeys with normal births needed a 1000-fold higher dose to establish intestinal infection than animals with stillbirths. This suggests that host susceptibility factors also play an important role in determining clinical outcomes during *L. monocytogenes* infection, and the use of an inbred animal model may be the easiest way to identify these genetic loci.

## Choice of *L. monocytogenes* strain

The choice of *L. monocytogenes* isolate used for oral challenge studies may also greatly alter the course of the infection. Barbour et al. infected BALB/c mice intragastrically with 66 different isolates and found that serovar 4b and 1/2a strains were most virulent, following the pattern observed in humans (Barbour et al., 2001). Most published studies have used serovar 1/2a strains such as EGD or 10403s, but the use of fresh clinical isolates may yield data that more accurately reflect particular types of human disease. Indeed, a recent microarray analysis suggested there may be identifiable differences between strains that cause primarily febrile gastroenteritis and those that cause more invasive disease (Laksanalamai et al., 2012). In support of this idea, Jensen et al. tested a strain (La111) that persistently colonized a fish processing plant and found that the bacteria were not more virulent *in vitro* and they did not persist in the intestines of orally infected pregnant guinea pigs. However, oral infection with strain La111 resulted in a significantly higher rate of infected fetuses than for other clinical isolates (Jensen et al., 2008a,b). In another example, Poulsen et al. found that pregnant mice were significantly more susceptible to oral infection with *L. monocytogenes* 2203, a strain isolated during an outbreak that affected primarily pregnant women (Macdonald et al., 2005; Poulsen et al., 2011).

“Murinized” strains of *L. monocytogenes* have also been developed to overcome the species barrier to receptor-mediated invasion of epithelial cells. The modified InlA (InlA^m^) expressed by these strains has a similar affinity for mouse E-cadherin as wild type InlA has for human E-cadherin (Wollert et al., 2007; Monk et al., 2010). Oral transmission of InlA^m^-expressing bacteria can be achieved with lower doses (10^6^–10^8^ CFU) and results in an infection course that closely mimics all phases of human disease (Bou Ghanem et al., 2012). However, a recent study showed that the modified InlA^m^ protein can bind both N-cadherin and E-cadherin, which results in enhanced uptake by villous M cells (Tsai et al., 2013). Thus, use of an InlA^m^-expressing strain may result in altered tropism of the bacteria compared to human infections.

## Route of inoculation

There are multiple ways to achieve oral transmission in lab animals. In one of the earliest approaches, Swiss mice were given *L. monocytogenes*-contaminated water for 24 h (Miller and Burns, 1970). However, since it was difficult to control the dose received, most investigators quickly adopted oral gavage techniques to place a defined inoculum directly into the stomach. Depending on the species being used, this can involve a blunted feeding needle pushed all the way to the stomach, or flexible tubing attached to a syringe.

Although the i.g. route provides more control over dose, it may have unintended consequences. First, when *L. monocytogenes* are administered in a liquid suspension, the majority of the bacteria transit quickly through the gut, and are shed in stool within 0.25–4 h (Hardy et al., 2004; Melton-Witt et al., 2012). Rapid passage may not allow enough exposure to the acidic pH and bile present in the upper GI tract for induction of the transcriptional changes that make *L. monocytogenes* more invasive in the gut (Conte et al., 2000). Intragastric inoculation also has the potential to cause minor physical trauma to the lining of the esophagus, particularly when a feeding needle is used, and this may facilitate direct bloodstream invasion that does not normally occur when *Listeria*-contaminated food is ingested. A comparative analysis of i.g. inoculation studies in mice indicates that some investigators found rapid spread to the spleen and liver in as little as 4 h (Lecuit et al., 2001; Czuprynski et al., 2003a; Gajendran et al., 2007; Wollert et al., 2007), while others found no systemic spread of *L. monocytogenes* until 48 h post-infection (Macdonald and Carter, 1980; Kursar et al., 2004; Monk et al., 2010). This suggests that i.g. inoculation is dependent on investigator-specific technique, and thus, does not yield highly reproducible results. Another solution to the problem of controlled dosage is to administer a bacterial solution directly into the mouth of the animal. For larger animals such as guinea pigs, it is relatively easy to slowly drip a solution into the oral cavity using a syringe (Melton-Witt et al., 2012). For smaller animals such as mice, Manohar et al. used a sterile bacterial inoculating loop to place a bacterial solution into the mouth (Manohar et al., 2001). More recently, Bou Ghanem et al. reported the development of a food borne model of listeriosis in mice (Bou Ghanem et al., 2012). This model has several advantages, including the ability to test the effect of different types of food or food storage conditions on transmissibility. Furthermore, the inoculation procedure is not invasive, does not cause physical trauma, and does not require specialized skills to perform, thereby lessening the chance of investigator-dependent variability.

It should be noted that there is some confusion in the literature regarding oral transmission routes, because not all reports describe the inoculation procedure and state simply that the animals were “orally infected.” Since the procedure used to achieve oral transmission of *L. monocytogenes* may alter the kinetics of bacterial invasion or dissemination, caution should be used when interpreting the results of a study that does not specify the technique used. Finally, the use of certain types of anesthesia for more invasive routes of inoculation may also alter susceptibility. Czuprynski et al. showed that sodium pentobarbital transiently enhanced the severity of infection in mice inoculated by the i.g. route, while isoflurane had no effect on infection rates (Czuprynski et al., 2003b; Sahaghian et al., 2009).

## Manipulations to enhance infectivity

One strategy that has been used to try to enhance oral transmission of *L. monocytogenes* in animal models is to neutralize stomach acid. Buffering an i.g. inoculum with sodium bicarbonate or providing only buffered drinking water significantly increased the number of *L. monocytogenes* recovered from the stomachs of mice 15 min. after inoculation (Saklani-Jusforgues et al., 2000). However, longer-term studies in animals are more inconclusive. Sodium bicarbonate pre-treatment did increase the severity of infection in neutropenic mice, but not in mice with normal levels of circulating neutrophils (Czuprynski and Faith, 2002; Czuprynski et al., 2002). Likewise, other studies found no difference in infectivity in mice pre-treated with cimetidine (Golnazarian et al., 1989) or in monkeys given a calcium carbonate solution 1 h prior to infection (Farber et al., 1991). However, Schlech et al. showed that pre-treatment with cimetidine did significantly lower the infectious dose in rats (Schlech et al., 1993). One reason for the different outcomes in these studies may be the endpoints used to determine infectivity. Neutralization of stomach acid may indeed prolong the initial survival of *L. monocytogenes*, however, colonization deeper in the intestines may be dependent on gene expression changes that occur following exposure to an acidic environment (O'Driscoll et al., 1996; Ramalheira et al., 2010). Thus, the small number of bacteria that survive passage through the unaltered stomach may be “gut-adapted” and better able to invade the gut mucosa.

The choice of delivery vehicle may also influence the infectivity of *L. monocytogenes* administered orally. In particular, several studies have examined the role of high fat content in enhancing the rate of establishing listeriosis. Bou Ghanem et al. found that food borne infection of mice was most consistent when bacteria suspended in melted butter, rather than saline, were used to contaminate small pieces of bread (Bou Ghanem et al., 2012). Likewise, Smith et al. showed that stillbirths in pregnant monkeys were more likely to occur when whipping cream, rather than whole or skim milk, was used as the delivery vehicle (Smith et al., 2003). These studies suggest that ingestion of a food product with a high fat content may promote bacterial survival or colonization in the gastrointestinal tract.

## Conclusions

Oral transmission of *L. monocytogenes* has not been widely used over the past few decades due to a high degree of innate resistance in many animals and significant phenotypic variability among infected animals. As summarized in this review, it can be difficult to compare previously published studies because of differences in bacterial strain choice, route of inoculation, and manipulations used to enhance infection. Recent technological advances, particularly for the mouse model, have greatly improved the efficiency of intestinal colonization following oral transmission, and the use of a standardized model would help to greatly advance the field.

### Conflict of interest statement

The author declares that the research was conducted in the absence of any commercial or financial relationships that could be construed as a potential conflict of interest.
